# Supplementary material for: A cis-carotene derived apocarotenoid regulates etioplast and chloroplast development
Source: eLife. 2020 Jan 31;9:e45310. doi: 10.7554/eLife.45310 (PMC6994220; doi:10.7554/eLife.45310)
Supplement: Supplementary file 1. [file elife-45310-supp1.docx]

| **Supplementary File 1. Immature *ccr2* tissues have an altered *cis*-carotene and xanthophyll composition.** | | | | | | | | | | |
| --- | --- | --- | --- | --- | --- | --- | --- | --- | --- | --- |
| **Genotype** | **Tissue** | **Age** | **Percentage xanthophyll composition** | | | | | | **Relative ratio** | |
|  |  |  | **lutein** | β**-c** | **zea** | **anth** | **viol** | **neo** | **phyt** | **pflu** |
| **WT** | **Rosette leaf** | **yng** | 50 | 26 | 0.0 | 0.5 | 10 | 13 | 0.00 | 0.00 |
|  | **Rosette leaf** | **old** | 48 | 27 | 0.1 | 0.6 | 11 | 13 | 0.00 | 0.00 |
|  | **Floral bud** | **yng** | 50 | 24 | 0.0 | 0.6 | 15 | 10 | 0.00 | 0.00 |
|  | **Floral bud** | **old** | 51 | 23 | 0.0 | 0.4 | 16 | 9 | 0.00 | 0.00 |
|  | **Maximum SD** | | 1 | 1 | 0.3 | 0.4 | 0 | 2 | ND | ND |
| ***ccr2*** | **Rosette leaf** | **yng** | 11 | 33 | 1.4 | 5.5 | 36 | 13 | 0.37 | 0.29 |
|  | **Rosette leaf** | **old** | 20 | 36 | 0.6 | 3.4 | 28 | 12 | 0.02 | 0.00 |
|  | **Floral bud** | **yng** | 12 | 34 | 1.3 | 4.2 | 38 | 11 | 1.55 | 0.59 |
|  | **Floral bud** | **old** | 17 | 34 | 1.0 | 3.5 | 33 | 12 | 0.76 | 0.38 |
|  | **Maximum SD** | | 1.2 | 0.6 | 0.2 | 0.7 | 0.7 | 0.3 | nd | nd |

Percentage of individual carotenoid levels relative to the total carotenoid content in different tissues from plants exposed to a 16 h photoperiod. Ratios of phytoene and phytofluene are relative to β-carotene. Data represent the average and maximum standard deviation (SD) for 2 biological replicates. Similar results were observed in independent experiments. Greyed highlighted values represent significant (t-test p<0.05) differences in immature younger (yng) relative to mature older (old) tissues. β-c; β-carotene**,** zea; zeaxanthin, anth; antheraxanthin, viol; violaxanthin, neo; neoxanthin, phyt; phytoene, pflu; phytofluene. nd; not determined.
