# Supplementary material for: A cis-carotene derived apocarotenoid regulates etioplast and chloroplast development
Source: eLife. 2020 Jan 31;9:e45310. doi: 10.7554/eLife.45310 (PMC6994220; doi:10.7554/eLife.45310)
Supplement: Supplementary file 2. [file elife-45310-supp2.docx]

**Supplementary File 2. D15 and *ziso* restore PLB formation in *ccr2* etiolated cotyledons**

| **Germplasm** | **Treatment** | **Number of etioplasts containing PLBs** | | | | |
| --- | --- | --- | --- | --- | --- | --- |
|  |  | **Total** | **PLB** | **Ratio (%)** | **SE (%)** | **Tukey Groups** |
| **WT** | **H_2_O** | 90 | 90 | 100 | 0.0 | **c** |
| **WT** | **EtOH** | 64 | 64 | 100 | 0.0 | **c** |
| **WT** | **D15** | 48 | 48 | 100 | 0.0 | **c** |
| ***ccr2*** | **H_2_O** | 63 | 0 | 0 | 0.0 | **a** |
| ***ccr2*** | **EtOH** | 72 | 0 | 0 | 0.0 | **a** |
| ***ccr2*** | **D15** | 71 | 61 | 85 | 2.5 | **b** |
| ***ziso1-4*** | **H_2_O** | 73 | 48 | 66 | 2.1 | **d** |
| ***ziso1-4*** | **D15** | 68 | 45 | 66 | 2.3 | **d** |
| ***ccr2 ziso1-4*** | **H_2_O** | 63 | 59 | 94 | 5.5 | **c** |
| ***ccr2 ziso-155*** | **H_2_O** | 79 | 76 | 95 | 5.0 | **c** |

PLB formation was examined in WT, *ccr2*, *ziso*, *ccr2* *ziso* and *ccr2 ziso-155* cotyledons 7 DAG in the dark. D15 (CCD inhibitor), EtOH (control solvent for dissolving D15) and/or H_2_O was added to the growth media treatments.
