# Supplementary material for: A cis-carotene derived apocarotenoid regulates etioplast and chloroplast development
Source: eLife. 2020 Jan 31;9:e45310. doi: 10.7554/eLife.45310 (PMC6994220; doi:10.7554/eLife.45310)
Supplement: Supplementary file 6. [file elife-45310-supp6.docx]

| **Supplementary File 6**. *det1* reduced carotenoids and caused *cis*-carotenes to accumulate in leaves and etiolated tissues | | | | | | | | | | | | |
| --- | --- | --- | --- | --- | --- | --- | --- | --- | --- | --- | --- | --- |
| **Genotype** | **Tissue** | ***cis*-carotenes**  **(μg/gfw)** | | | **Carotenoids**  **(μg/gfw)** | | | | | | | **Chlorophyll (μg/gfw)** |
|  |  | **phyt** | **pflu** | **3ζ-C** | **neo** | **viol** | **anth** | **lut** | **zea** | **β-c** | **total** |  |
| **WT** | **leaves** | nd | nd | nd | 37 | 47 | 2 | 151 | nd | 96 | 333 | 1470 |
| ***det1-1*** |  | trace | trace | nd | 15 | 32 | 0 | 70 | nd | 52 | 170 | 728 |
| ***det1-154*** |  | trace | trace | nd | 19 | 35 | 0 | 73 | nd | 61 | 188 | 818 |
|  | **SE** |  |  |  | 2 | 8 | 1 | 9 | 0 | 6 | 22 | 96 |
| **WT** | **cotyledons** | nd | nd | nd | 1.0 | 6.5 | 0.7 | 17.5 | nd | 0.8 | 26.5 | nd |
| ***det1-1*** |  | 0.19 | 0.15 | 0.08 | nd | 1.8 | nd | 6.8 | nd | 0.4 | 8.8 | nd |
|  | **SE** | 0.00 | 0.00 | 0.01 | 0.1 | 0.7 | 0.1 | 1.7 |  | 0.1 | 2.6 |  |

Absolute carotenoid and chlorophyll levels in young emerging leaves (16 h photoperiod) and etiolated cotyledons (7-d-old). Data represent the average and maximum standard error (SE; n=3 to 12 biological replicates). Similar results were observed in independent experiments. Grey shading denote significant differences compared to WT (ANOVA; p<0.05). β-c; β-carotene**,** zea; zeaxanthin, anth; antheraxanthin, viol; violaxanthin, neo; neoxanthin, phyt; phytoene, pflu; phytofluene, 3ζ-C; tri-cis-ζ-carotene, nd; not detectable.
