# Supplementary material for: A cis-carotene derived apocarotenoid regulates etioplast and chloroplast development
Source: eLife. 2020 Jan 31;9:e45310. doi: 10.7554/eLife.45310 (PMC6994220; doi:10.7554/eLife.45310)
Supplement: Supplementary file 7. [file elife-45310-supp7.docx]

| **Supplementary File 7. Primer sequences used for qPCR and *ccr2 det154* characterisation** | | | |
| --- | --- | --- | --- |
| **PCR primers** | **Purpose or gene** | **Sequence** |  |
| det1-short-F | Amplifies *det1* exon 3-5 junction | GAATGAAGAATCAGATAACGTAATGgttcag |  |
| det1-short-R | Amplifies *det1* exon 6-7 junction | TGGTAAAGATCTTCTGCTGAGTTCTGA |  |
| det1-long-F | Amplifies *det1* exon 3-4 | TGAAGAATCAGATAACGTAATGAGAGTTC |  |
| det1-long-R | Amplifies *det1* exon 4-5 | TGATCAGCACTTCTTGTTACCCCA |  |
| PP2A-F | protein phosphotase 2A | CTTCGTGCAGTATCGCTTCTC |  |
| PP2A-R | protein phosphotase 2A | ATTGGAGAGCTTGATTTGCG |  |
| PORA-F | protochlorophyllide oxidoreductase a | TTTCGGAGCAAAGCAAAGC |  |
| PORA-R | protochlorophyllide oxidoreductase a | TTTGTGACTGATGGAGTTGAAG |  |
| LHCB2-F | light-harvesting chlorophyll b-binding 2 | CAGCTATCCAACAATCCTCCTTCG |  |
| LHCB2-R | light-harvesting chlorophyll b-binding 2 | TTCTCCGAGAATGGTCCCAAGTAC |  |
| HY5-F | elongated hypocotyl-5 | GAGAAAGAGAACAAGCGGCTGAAG |  |
| HY5-R | elongated hypocotyl-5 | AGCATCTGGTTCTCGTTCTGAAGA |  |
| PIF3-F | phytochorme interacting factor 3 | TTGGCTCGGGTAATAGTCTCGATG |  |
| PIF3-R | phytochorme interacting factor 3 | CCTGCTTCCTTTCTTCCATCTCCT |  |
|  |  |  |  |
